# Short-term effect of sacubitril/valsartan on endothelial dysfunction and arterial stiffness in patients with chronic heart failure

**DOI:** 10.3389/fphar.2022.1069828

**Published:** 2022-12-05

**Authors:** Velia Cassano, Giuseppe Armentaro, Marcello Magurno, Vincenzo Aiello, Francesco Borrello, Sofia Miceli, Raffaele Maio, Maria Perticone, Alberto M. Marra, Antonio Cittadini, Marta L. Hribal, Francesco Andreozzi, Giorgio Sesti, Angela Sciacqua

**Affiliations:** ^1^ Department of Medical and Surgical Sciences, University Magna Græcia of Catanzaro, Catanzaro, Italy; ^2^ Rheumatology Unit, Department of Clinical Medicine and Surgery, University Federico II, Naples, Italy; ^3^ Division of Cardiology and Intensive Cardiac Care Unit, Pugliese-Ciaccio Hospital, Catanzaro, Italy; ^4^ IRCCS SDN, Naples, Italy; ^5^ Department of Translational Medical Sciences, University Federico II of Naples, Naples, Italy; ^6^ Research Center for the Prevention and Treatment of Metabolic Diseases, University of Catanzaro, Catanzaro, Italy; ^7^ Department of Clinical and Molecular Medicine, Sapienza University of Rome, Rome, Italy

**Keywords:** heart failure, endothelial dysfunction, arterial stiffness, sacubitril/vasartan, oxidative stress

## Abstract

Heart failure (HF) is associated to endothelial dysfunction that promotes the increase of arterial stiffness thus augmenting myocardial damage. Sacubitril/Valsartan is used in the treatment of HF reduced ejection fraction (HFrEF) and has been proven effective in reducing cardiovascular disease (CVD) progression and all-cause mortality. The aim of this study was to evaluate the effect of Sacubitril/Valsartan on endothelial dysfunction, arterial stiffness, oxidative stress levels and platelets activation in patients with HFrEF, at baseline and after 6 months of treatment. We enrolled 100 Caucasian patients. Endothelial function was evaluated by the reactive hyperemia index (RHI) and arterial stiffness (AS) by the measurement of carotid-femoral pulse wave velocity (PWV), augmentation pressure (AP) and augmentation index (AI). At baseline, among enrolled outpatients, 43% showed a NYHA class II and 57% a NYHA class III. At 6 months, there was a significant improvement of several hemodynamic, clinical and metabolic parameters with a significant reduction in oxidative stress indices such as 8-isoprostane (*p* < 0.0001) and Nox-2 (*p* < 0.0001), platelets activity biomarkers such as sP-selectin (*p* < 0.0001) and Glycoprotein-VI (*p* < 0.0001), and inflammatory indices. Moreover, we observed a significant improvement in arterial stiffness parameters and in endothelial function indices. Our study demonstrated that 6 months treatment with Sacubitril/Valsartan, in patients with HFrEF, improves endothelial dysfunction and arterial stiffness, by reducing oxidative stress, platelet activation and inflammation circulating biomarkers, without adverse effects.

## 1 Introduction

Heart failure (HF) is a widespread health problem and represents a major cause of cardiac morbidity and mortality representing the second cause of hospitalization in subjects >65 years old, despite optimal treatment (Tavazzi et al., 2013; Armentaro et al., 2021). Among affected patients, HF with reduced ejection fraction (HFrEF) represents about 50% of cases (Iorio et al., 2019).

Notably, endothelial dysfunction is a frequent condition in patients affected by HF and in this setting it predicts major cardiovascular (CV) events and mortality. Endothelial dysfunction is characterized by reduced bioavailability of nitric oxide (NO), in response to increased reactive oxygen species (ROS) production, with an imbalance between vasoconstrictor and vasodilator factors, predisposing to a pro-atherogenic and pro-trombotic phenotype, characterized by leukocyte adhesion, mitogenesis, platelet activation and vascular inflammation (Dhananjayan et al., 2016; Alem, 2019). Endothelial dysfunction may also result from abnormal electrical property and the changed expression profile of ion channels (Fan et al., 2022a).

It is known that inflammation and oxidative stress, characterized by increased 8,12-isoprostane F (2α) concentration, low endothelium-bound extracellular superoxide dismutase activity and increased endothelium-bound xanthine oxidase (XO) activity and cytokines production, play a central role in pathogenesis of both endothelial dysfunction and HF (Bulua et al., 2011; Odegaard et al., 2016; Alem, 2019; Cassano et al., 2022). Moreover, endothelial dysfunction attracts circulating inflammatory cells through Intercellular adhesion molecules (ICAMs) and inflamed endothelium interacts with cardiomyocytes and platelets (El-Battrawy et al., 2016b). In addition, increased ROS levels are one of the causes of the redox imbalance through many molecular mechanisms, as for example augmented secretion of proinflammatory cytokines (El-Battrawy et al., 2017) (Fan et al., 2022b).

Arterial stiffness, resulting from reduced elasticity of the arterial wall, increases with age and different cardiometabolic abnormalities promoting endothelial dysfunction and representing an independent predictor of CV events and HF (Husmann et al., 2015; Cassano et al., 2020). According with this, recently, many studies investigated mechanistic pathways of endothelial dysfunction and arterial stiffness in HF with the aim to provide the rationale for new drugs development and to improve the application of existing therapy (Alem, 2019).

It is known that, arterial stiffness and endothelial dysfunction are strongly interconnected recognizing similar pathophysiological mechanisms. In particular, arterial stiff-ness is correlated with increased activity of angiotensin II, which activates matrix metalloproteinases (MMP) and pro-inflammatory cytokines including tumor necrosis factor alpha (TNF-α) and interleukin-6 (IL-6). Inflammatory cytokines promote the production of C reactive protein (CRP) by vascular smooth muscle cells, which increases vascular inflammation and reduces endothelium-dependent vasodilation (Park and Lakatta, 2012). The increased vascular inflammation promotes smooth muscle cells proliferation, vascular fibrosis and impair endothelial vasodilatation, which leads to worsening of arterial stiffness, the last in turn, by inducing a reduction in vascular shear stress, may further reduce NO bioavailability.

Sacubitril/Valsartan is the first angiotensin receptor-neprilysin inhibitor (ARNI) acting by reducing the degradation of natriuretic peptides and blocking selective AT1-receptor with positive effects on CV system (Vardeny et al., 2014). In HFrEF outpatients, the PARA-DIGM-HF study demonstrated that Sacubitril/Valsartan was associated with a lower risk of composite endpoint of CV mortality or first hospitalization for HF compared to enalapril, during a twenty-seven months median of follow-up. In addition, in patients with acute HF, Sacubitril/Valsartan resulted more efficacious than enalapril in reducing both N-terminal pro-brain natriuretic peptide (NT pro-BNP) levels and rehospitalization and CV death, in an exploratory analysis (Velazquez et al., 2019). Data from real life showed that Sacubitril/Valsartan is associated with improvement in cardiac remodelling, New York Heart Association (NYHA) functional class and exercise tolerance with consequently hospital readmissions reduction (Armentaro et al., 2021). In addition, a study conducted by Abumayyaleh demonstrated improvement in cardiac valvular insufficiency, after 24 months of treatment with sacubitril/valsartan in patients with HFrEF (Abumayyaleh et al., 2022).

Moreover, a post hoc analysis from PARADIGM-HF demonstrated that subjects with type 2 diabetes mellitus (T2DM) in treatment with Sacubitril/Valsartan presented an improved glycaemic profile, compared to patients in treatment with enalapril, after a follow-up of 12 months (Seferovic et al., 2017). In addition, results from a post-hoc analysis of PARAGON-HF study demonstrated that subjects with HF and preserved ejection fraction (HFpEF), in treatment with Sacubitril/Valsartan, presented significant reduction in triglycerides and increase in HDL cholesterol. Therefore, the inhibition of renin angiotensin aldosterone system (RAAS) and neprilysin by Sacubitril/Valsartan results also in metabolic improvement as recently demonstrated in real life setting (Armentaro et al., 2022). But the benefits of Sacubitril/Valsartan are not limited to the treatment of HFrEF through modulation of the RAAS and enhancement of natriuretic peptides, an anti-arrhythmic effect is also observed due to the reduction of pro-arrhythmic remodelling and/or a direct anti-arrhythmic effect on cardiomyocytes (Sutanto et al., 2021) (Abumayyaleh et al., 2021). However, there are controversial data regarding the efficacy of sacubitril/valsartan in the treatment of ventricular tachycardia and/or ventricular fibrillation. A study conducted by El-Battrawy et al. 2019c demonstrated in HFrEF patinets, that treatment with sacubitril/valsartan does not reduce the risk of ventricular tachyarrhythmias, after 12 months of follow- up. By contrast, recently, a study showed a significant reduction in ventricular arrhythmias in HFrEF patients, in treatment with sacubitril/valsartan (El-Battrawy et al., 2019a).

Taken together, all these findings could suggest a possible direct protective effect of Sacubitril/Valsartan on CV system by modulating inflammation and oxidative stress. According with this, a recent study conducted by Qing Ge et al. (2019) in an animal model, demonstrated that Sacubitril/Valsartan could protect against diabetic cardiomyopathy (DCM) through the inhibition of inflammatory mediators, oxidative stress and apoptosis, without adverse effects.

By the way, in literature no study investigated the possible effect of treatment with Sacubitril/Valsartan on endothelial dysfunction and arterial stiffness in patients with HF.

With the aim to better understand pathophysiological mechanisms underlying cardiovascular protective effects of Sacubitril/Valsartan, and in agreement with evidence al-ready in the literature, the aim of the present study was to evaluate the effect of Sacubitril/Valsartan on endothelial dysfunction and arterial stiffness in patients with HFrEF evaluated at baseline and after 6 months of treatment. Moreover, we evaluated the effects of Sacubitril/Valsartan on oxidative stress levels and platelets activation and the correlation with arterial stiffness and endothelial dysfunction.

## 2 Materials and methods

### 2.1 Study population

We performed a prospective, one-center study, analysing all clinical, laboratory, and vascular parameters of 100 consecutive HFrEF Caucasian subjects (82 men and 18 women, mean age 69.7 ± 7.7) followed at the Catanzaro University Hospital, Geriatric Department. Patients’ inclusion criteria were age >18 years, left ventricular ejection fraction (LVEF) < 35%, NYHA class II or III, symptomatic patients despite optimized treatment with stable doses of angiotensin- converting enzyme inhibitor (ACE-I) or angiotensin receptor blocker (ARB) for at least four weeks. All enrolled patients underwent to Sacubitril/Valsartan treatment according to International Guidelines recommendations (McDonagh et al., 2021). No patient presented clinical history of hepatic impairment (Child-Pugh Class C), severe renal disease [estimated-glomerular filtration rate (e-GFR) <30 ml/min/1.73 m^2^], angioedema or side effects due to ACE-I or ARB. Moreover none had systolic blood pressure (SBP) < 100 mmHg or potassium levels >5.4 mmol/L. Women were exclude if pregnant or breastfeeding. In addition to previous treatments, eligible patients for Sacubitril/Valsartan discontinued ACE-I (at least 36 h before) or ARB, and received initial dosages of 24/26 mg or 49/51 mg bid according to clinical conditions. Moreover, Sacubitril/Valsartan dosage was increased every 2–4 weeks up to the maximum tolerated dose, as recommended.

In all patients a complete medical history and careful physical examination were performed.

Ethics Committee Regione Calabria “Area Centro” approved the protocol (code protocol number 2012.63). All participants gave their informed consent for personal data treatment and all investigations were made according to Helsinki Declaration.

All clinical evaluations and laboratory tests were performed at baseline (time T0) and after 6all patients a complete medicalmonths of treatment (time T6).

### 2.2 Blood pressure measurements

After 5 min of rest, clinical blood pressure (BP) measurement were obtained with an aneroid sphygmomanometer in the left arm of supine patients. The diagnosis of arterial hypertension was carried out in all subjects with SBP >140 mmHg and/or diastolic BP (DBP) > 90 mmHg according to current guidelines (Williams et al., 2018). The difference between systolic and diastolic BP values identified the pulse pressure (PP) measurement.

### 2.3 Laboratory determination

After at least 12 h fasting, the laboratory measurements were performed. The glucose oxidation method (Beckman Glucose Analyzer II; Beckman Instruments, Milan, Italy) was utilized to measure plasma glucose, and a chemiluminescence-based assay (Roche Diagnostics) for plasma insulin determination. An enzymatic method (Roche Di-agnostics GmbH, Mannheim, Germany) was used to detect total, low and high-density lipoprotein (LDL, HDL) cholesterol and triglyceride concentrations. Serum creatinine was determined using a Roche Creatinine Plus assay (Hoffman-La Roche, Basel, Switzerland) on a clinical chemistry analyser (Roche/Hitachi Modular Analytics System, P Module). Renal function was evaluated by calculating the estimate glomerular filtration rate (e-GFR) using the CDK-EPI equation (Levey et al., 2009). An enzyme-linked immunosorbent assay (Elecsys proBNP assay, Roche Diagnostics) was utilized to assess N-terminal pro-brain natriuretic peptide (NT-proBNP) levels. Serum sodium and potassium levels were obtained by indirect potentiometry (Cobas, Roche) and high sensitive C-reactive protein (hs-CRP) by an automated instrument (Cardio-Phase1hsCRP), Milan, Italy).

#### 2.3.1 Serum levels of oxidative stress and platelets activation biomarkers

Blood samples were taken in tubes with separator gel and centrifuged at 4,000 rpm for 15 min to obtain serum samples. In order to prevent oxidation samples were stored at −80°C in the presence of 0.005% butylated-hydroxy-toluene (BHT) (10 µL of 5 mg/ml solution in ethanol per 1 ml sample). Specific ELISA kits were employed to detected serum levels of 8-isoprostane (ELISA kit Cayman Chemical Michigan, United States); NAPDH Oxidase 2 (Nox-2), human glycoprotein VI (GPVI) and Sp-selectin (all from ELISA kit MyBioSource, California, United States). Values of 8-isoprostane were expressed as pg/mL; the lower detection limit of the assay was 0.8 pg/ml and interassay coefficient of variation (CV) was <9.6%. Values of Nox-2 were expressed as nmol/L, the lower detection limit of the assay was 0.25 nmol/L, the intra-assay CV was <9%, the interassay CV was <11%.

Values of GPVI were expressed in pg/ml, the lower detection limit of the assay was 46.88 pg/ml, the intra-assay CV was <8% and the interassay CV was <10%. Finally, Sp-selectin concentrations were expressed in ng/ml, the kit had a lower detection limit of 15 ng/ml, the intra-assay was CV <10% and the interassay CV was <15%.

#### 2.3.2 Cytokines determinations

The Biochip Array (Randox Labs) was used for the assay of serum cytokines (IL-6, TNF-α) (Cassano et al., 2021), through chemiluminescence the immune reaction was revealed, the signal emitted being directly related to the increase in cytokine levels. The signal was detected using digital imaging technology, and the concentration of the analyte was calculated from the calibration curve by an Evidence Investigator systems analyzer (Randox Labs, United Kingdom) with dedicated software.

### 2.4 Arterial stiffness evaluation

We used high-fidelity applanation tonometry (Millar) and pressure wave analysis computer software (Sphygmocor™), which is a validated system, to measure arterial stiffness. Automatic, non-invasive recording of supine brachial blood pressure of the dominant arm after a 30-min rest was used for pressure calibration (Dinamap Com-pact T; Johnson & Johnson Medical Ltd., Newport, United Kingdom). Within 10 min, 5 BP measurements were taken and the average of the last 3 was used for calibration. The radial artery of the dominant arm with the wrist slightly hyperextended was used to record pres-sure waves representing the average of individual pressure waves recorded consecutively for 8 s. Only pressure waves with peak and background pressure variation of individual waves <5% were used. Using an integrated generalised transfer function, the central pressure wave was automatically derived from the radial pressures. Pressure wave measurements were also obtained at the level of the right carotid artery, resulting in a more precise central augmentation index (AI) (Cassano et al., 2020). In addition, central waveforms were analysed to obtain the time at the peak/shoulder of the first (T1) and second (T2) pressure wave component during systole. The height of the outgoing pressure wave (P1) was identified as the pressure at the peak/shoulder of T1 while the height of the reflected pressure wave (P2) as the pressure at the peak/shoulder of T2, either in absolute terms or as a percentage of the ejection duration. The difference between P2 and P1 was used to define the augmentation pressure (AP), while AI was defined as [AP/PP] × 100. The carotid and femoral pressure waveforms were used to define the aortic pulse wave velocity (PWV). From the foot-to-foot time difference between the carotid and femoral waveforms, the carotid-to-femur transit time (ΔT) was calculated. The distance between the surface marks of the sternal notch and femoral artery was used to estimate the path length between the carotid and femoral arteries (L), and the PWV calculated as L/ΔT.

### 2.5 Endothelial function evaluation

A semi-plethysmographic method with digital pulse volume amplitude (PVA) measurement was used to assess endothelial function with patients in a supine position in a quiet, temperature-controlled environment set at 22°C. PVA was measured using the peripheral arterial tonometer in the fingertip of the index finger (Itamar-Medical, Caesarea, Israel) both at rest and during reactive hyperemia (RH). The peripheral arterial tonometer consists of a finger-mounted probe that encloses the fingertip with an inflatable, pressurised air cushion that is electronically controlled and confined in a rigid outer shell. Pressure changes within the probe accompanying PVA changes in the fingertip were trans-mitted to a personal computer where the signal was band-filtered (0.3–30 Hz), displayed, amplified and stored. From the release of an inflated upper arm BP cuff above the SBP for 5 min, the RH was obtained. The ratio of the mean PVA over a 1-min time interval from 1 min after cuff deflation (RH) to the mean PVA assessed for 1 min before cuff inflation (baseline) defines the digital PVA-RH. Any deviations in signal magnitude due to systemic factors were assessed by constant evaluation of the PVA of the index finger of the other non-ischemic hand (which was not subjected to RH) throughout the study (Cassano et al., 2021).

### 2.6 Statistical analysis

Continuous variables were expressed as mean ± standard deviation (SD) (normally distributed data) or as median and interquartile range (IQR) (non-normally distributed data). Categorical data were expressed ass percentage. For all continuous variables, comparisons between baseline (T0) and post-treatment values (T6) were performed using paired Student’s t test for normally distributed data or Wilcoxon’s test for non-normally distributed data. For categorical data comparison was conducted by chi-square test. All variables which deviate from the normal distribution were log-transformed (ln) before correlational analysis. A linear regression analysis was performed to assess the relation-ship between variation in arterial stiffness (PWV) and endothelial function (RHI) indices, expressed as Δ of variation between baseline and follow-up (ΔT0–6) and the variation of metabolic, inflammatory, oxidative stress and platelets activation covariates that significantly changed after the treatment (expressed as ΔT0–6). Variables reaching statistical significance were inserted in a stepwise multivariate linear regression model to assess the magnitude of their individual effect on ΔPWV and ΔRHI. Moreover, a linear regression analysis was performed to assess the relationship between variation in oxidative stress indices (expressed as ΔT0–6) and different covariates (expressed as ΔT0–6) and to evaluate the relationship between variation in platelets activation and oxidative stress bi omarkers (expressed as ΔT0–6). Differences were assumed to be significant at *p* < 0.05. All comparisons were performed using SPSS 20.0 statistical software for Windows (SPSS, Inc, Chicago, IL).

## 3 Results

### 3.1 Study population

Of the 100 outpatients evaluated, 82% were males, 13% active smokers, 43% had NYHA class II and 57% had NYHA class III. Tables 1, 2 show demographic, clinical characteristics and drug therapy of the whole study population and in agreement with gender, at baseline.

**TABLE 1:** Demographic, clinical characteristics and drug therapy of the study population at baseline.

	Whole population (*n* = 100)
Gender, m/f (%)	82/18 (82/18)
Age, years	69.7 ± 7.7
Smokers, n (%)	13 (13)
Ischemic Heart Disease, n (%)	76 (76)
Valvular Disease, n (%)	30 (30)
AF, n (%)	32 (32)
T2DM, n (%)	66 (66)
Arterial hypertension, n (%)	84 (84)
COPD, n (%)	35 (35)
Dyslipidemia, n (%)	87 (87)
CKD, n (%)	27 (27)
Therapies	
ACEi/ARBs, n (%)	100 (100)
MRAs, n (%)	51 (51)
Statins, n (%)	78 (78)
Beta blockers, n (%)	99 (99)
Antiplatelet drugs, n (%)	54 (54)
Oral anticoagulant, n (%)	32 (32)
Diuretics, n (%)	99 (99)
OADs, n (%)*	66 (100)
Insulin therapy, n (%)*	25 (38)
Metformin, n (%)*	64 (97)
GLP-1 RAs, n (%) *	13 (20)

Abbreviation: AF, atrial fibrillation; T2DM, Type 2 diabetes mellitus; COPD, chronic obstructive pulmonary disease; CKD, chronic kidney disease; ACE, angiotensin-converting enzyme inhibitors; ARBs, angiotensin receptor blockers; MRAs, mineral receptor antagonists; OAD, oral antidiabetic drugs; GLP-1 RA, glucagon-like peptide 1 receptor agonists. *, only diabetic patients.

**TABLE 2:** Demographic, clinical characteristics and drug therapy of the study population, according to the gender, at baseline.

	Male population (*n* = 82)	Female population (*n* = 18)
Age, years	69.6 ± 7.7	70.4 ± 7.8
Smokers, n (%)	10 (12.1)	3 (16.7)
Ischemic Heart Disease, n (%)	67 (82.0)	9 (50.0)
Valvular Disease, n (%)	21 (25.6)	9 (50.0)
AF, n (%)	24 (29.2)	8 (44.4)
T2DM, n (%)	54 (66.0)	12 (66.7)
Arterial hypertension, n (%)	69 (84.1)	15 (83.3)
COPD, n (%)	30 (36.6)	5 (27.8)
Dyslipidemia, n (%)	71 (86.6)	16 (88.9)
CKD, n (%)	20 (24.4)	7 (38.9)
Therapies		
ACEi/ARBs, n (%)	82 (100)	18 (100)
MRAs, n (%)	41 (50.0)	10 (55.6)
Statins, n (%)	65 (79.2)	13 (72.2)
Beta blockers, n (%)	81 (98.8)	18 (100)
Antiplatelet drugs, n (%)	43 (52.4)	11 (61.1)
Oral anticoagulant, n (%)	24 (29.3)	8 (44.4)
Diuretics, n (%)	81 (98.8)	18 (100)
OADs, n (%)*	54 (100)	12 (100)
Insulin therapy, n (%)*	21 (38.9)	4 (33.3)
Metformin, n (%)*	52 (96.2)	12 (100)
GLP-1 RAs, n (%) *	10 (20.0)	3 (25.0)

Abbreviation: AF, atrial fibrillation; T2DM, Type 2 diabetes mellitus; COPD, chronic obstructive pulmonary disease; CKD, chronic kidney disease; ACE, angiotensin-converting enzyme inhibitors; ARBs, angiotensin receptor blockers; MRAs, mineral receptor antagonists; OAD, oral antidiabetic drugs; GLP-1 RA, glucagon-like peptide 1 receptor agonists. *, only diabetic patients.

The main aetiologies for HF were ischemic heart disease in 76% of the study group, valvulopathies in 30% and arterial hypertension in 84%. Considering the associated comorbidities, atrial fibrillation was present in 32% of the study population, 66% of the patients presented type 2 diabetes mellitus (T2DM), 35% showed chronic obstructive pulmonary disease (COPD) and 87% had dyslipidaemia. The different drug classes taken by the study patients are assumed in Table 1. At baseline 51% of patients were taking mineral receptor agonists (MRA), whose intake decreased by 36% at 6 months follow-up (*p* = 0.032). At baseline, all patients with T2DM were in treatment with oral antidiabetic drugs (OADs), at 6 months follow-up 87% were still taking OADs. In addition, at baseline 38% of diabetic patients were treated with insulin whereas at 6 months of follow-up 23% continued insulin treatment (*p* = 0.038). At baseline 35% diabetic patients were treated with OADs and insulin concomitantly, while at 6 months only 23%. Moreover, among diabetic patients, only 20% were in treatment with glucagon-like peptide 1 receptor agonist (GLP-1 RA) at baseline. On the other hand, regarding the intake of beta-blockers, there was no change in their use. All 100 enrolled patients completed the follow-up.

At baseline, 71% of the population started the lowest dose of Sacubitril/Valsartan (24/26) and 29% of the patients the intermediate dose (49/51). After 6 months of follow-up, there was a significant improvement of the functional status, thus 41% of patients shifted to NYHA class I, 51% of patients showed a NYHA class II and 8% remained in NYHA class III. After 6 months of follow-up, 16% of patients were taking the lowest dose of Sacubitril/Valsartan (24/26), 61% of the patients the intermediate dose (49/51) and 23% the highest dose (97/103).

### 3.2 Evaluation of study biomarkers over time

The main anthropometric and biochemical parameters of the study population at baseline and after the 6-month treatment period are reported in Table 3. After 6 months of treatment with Sacubitril/Valsartan, there was significant improvement in hemodynamic and clinical parameters, in particular a statistically significant reduction in heart rate (HR) (80.6 ± 9.9 vs. 74.5 ± 6.7 bpm, *p* < 0.0001), respiratory rate (RR) (17.7 ± 2.3 vs. 16.2 ± 1.5 breath/min, *p* < 0.0001), SBP (124.4 ± 10.2 vs. 121.3 ± 10.3, *p* < 0.0001), DBP (71.4 ± 6.2 vs. 68.2 ± 6.2, *p* < 0.0001), NT-ProBNP (from 3,163 (1,238–4,123) to 2,845 (789–3,179.25) pg/ml, *p* < 0.0001), sodium (139.8 ± 1.9 vs. 139.3 ± 1.5 mmol/L, *p* = 0.015), creatinine (1.1 ± 0.4 vs. 1.0 ± 0.3 mg/d, *p* < 0.0001); on the contrary, e-GFR (69.1 ± 18.3 vs. 104.4 ± 28.9 ml/min/m2, *p* < 0.0001), potassium (4.3 ± 0.3 vs. 4.5 ± 0.4 mmol/L, *p* = 0.001), haemoglobin (Hb) (11.6 ± 1.1 vs. 12.6 ± 1.6 g/dl, *p* < 0.0001) and insulin-like growth factor-1 (IGF-1) (90.8 ± 20.7 vs. 95.0 ± 20.7; *p* = 0.005) significantly increased. Moreover there was a statistically significant improvement in the glycometabolic profile as shown in Table 3. Regarding the inflammatory profile, there was a statistically significant reduction in hs-CRP (7.6 ± 0.4 vs. 6.8 ± 0.4, *p* < 0.0001), IL-6 (7.5 (7.0–9.5) vs. 5.6 (5.0–7.5); *p* < 0.0001), TNF-α (5.0 (4.1–5.7) vs. 4.0 (3.2–4.8); *p* < 0.0001), indicating an improvement in the inflammatory state, after 6 months treatment with Sacubitril/Valsartan.

**TABLE 3:** Anthropometric, hemodynamic, and biochemical characteristics of the study population, at baseline and after 6 months follow-up.

	Baseline	Follow-up (6 months)	*P*
Weight, kg	96.7 ± 14.8	94.4 ± 13.9	<0.0001
BMI, kg/m^2^	33.7 ± 4.1	32.6 ± 4.8	<0.0001
SBP, mmHg	124.4 ± 10.2	121.3 ± 10.3	<0.0001
DBP, mmHg	71.4 ± 6.2	68.2 ± 6.2	<0.0001
PP, mmHg	53.0 ± 10.2	53.1 ± 10.1	0.743
HR, bpm	80.6 ± 9.9	74.5 ± 6.7	<0.0001
RR, breath/min	17.7 ± 2.3	16.2 ± 1.5	<0.0001
Hb, g/dl	11.6 ± 1.1	12.6 ± 1.6	<0.0001
Hct, %	32.4 ± 3.1	37.2 ± 4.8	<0.0001
RBC, µ/L	3.9^10^6^ ± 0.7	4.2^10^6^ ± 0.5	<0.0001
WBC, µ/L	5.8 ± 1.1	6.1 ± 1.1	0.015
MCHC, g/dl	32.5 ± 1.6	32.3 ± 1.3	0.182
MCH, pg	29.4 ± 4.4	29.6 ± 4.1	0.539
MCV, fl	88.7 ± 4.0	88.7 ± 3.5	0.989
PLT, U/µL	205.1 ± 49.1	204.2 ± 49.1	0.878
K, mmol/L	4.3 ± 0.3	4.5 ± 0.4	0.001
Na, mmol/L	139.8 ± 1.9	139.3 ± 1.5	0.015
FPG, mg/dl	130.7 ± 35.8	117.8 ± 33.7	<0.0001
FPI, µU/ml	22.7 ± 3.7	18.6 ± 4.0	<0.0001
HOMA, (mmol/L*[μU/mL]/22.5)	7.3 ± 2.4	5.4 ± 2.0	<0.0001
LDL cholesterol, mg/dl	82.7 ± 28.0	79.1 ± 27.4	0.167
HDL cholesterol, mg/dl	40.2 ± 8.5	41.2 ± 8.4	0.323
TRG, mg/dl	176.9 ± 65.2	153.7 ± 48.0	<0.0001
Creatinine, mg/dl	1.1 ± 0.4	1.0 ± 0.3	<0.0001
e-GFR, ml/min/1.73m^2^	69.1 ± 18.3	104.4 ± 28.9	<0.0001
HbA1c, %	7.1 ± 1.1	6.7 ± 1.1	<0.0001
NT-ProBNP, pg/ml	3,163 (1,238–4,123)	2,845 (789–3,179.25)	<0.0001
Uric acid, mg/dl	6.7 ± 0.7	6.2 ± 0.9	<0.0001
hs-CRP, mg/L	7.6 ± 0.4	6.8 ± 0.4	<0.0001
IGF-1, ng/ml	90.8 ± 20.7	95.0 ± 20.7	0.005
IL-6, pg/ml	(7.5 (7.0–9.5)	5.6 (5.0–7.5)	<0.0001
TNF-α, pg/ml	5.0 (4.1–5.7)	4.0 (3.2–4.8)	<0.0001

Abbreviations: BMI, body mass index; SBP, systolic blood pres sure; DBP, diastolic blood pressure; PP, pulse pressure; HR, heart rate; RR, respiratory rate; Hb, haemoglobin; Hct, ematocrite; RBC, red blood cells; WBC, white blood cells; MCHC, mean corpuscular haemoglobin concentration; MCH, mean corpuscolar haemoglobin; PLT, platelets; K, potassium; Na, Sodium; FPG, fasting plasma glucose; FPI, fasting plasma insulin; HbA1c, glycated haemoglobin; HOMA, homeostasis model assessment; LDL, low density lipoprotein; HDL, high density lipoprotein; TRG, triglycerides; e-GFR, estimated glomerular filtration rate; NT-proBNP, N-terminal pro-brain natriuretic peptide; IGF-1, insulin-like growth factor-1; hs-CRP, highly sensitive c-reactive protein; IL-6, interleukin-6; TNF-α, Tumour necrosis factor *α*. Data are mean ± SD. a, Overall differences between baseline and follow-up (Student t-test).

**FIGURE 1:**
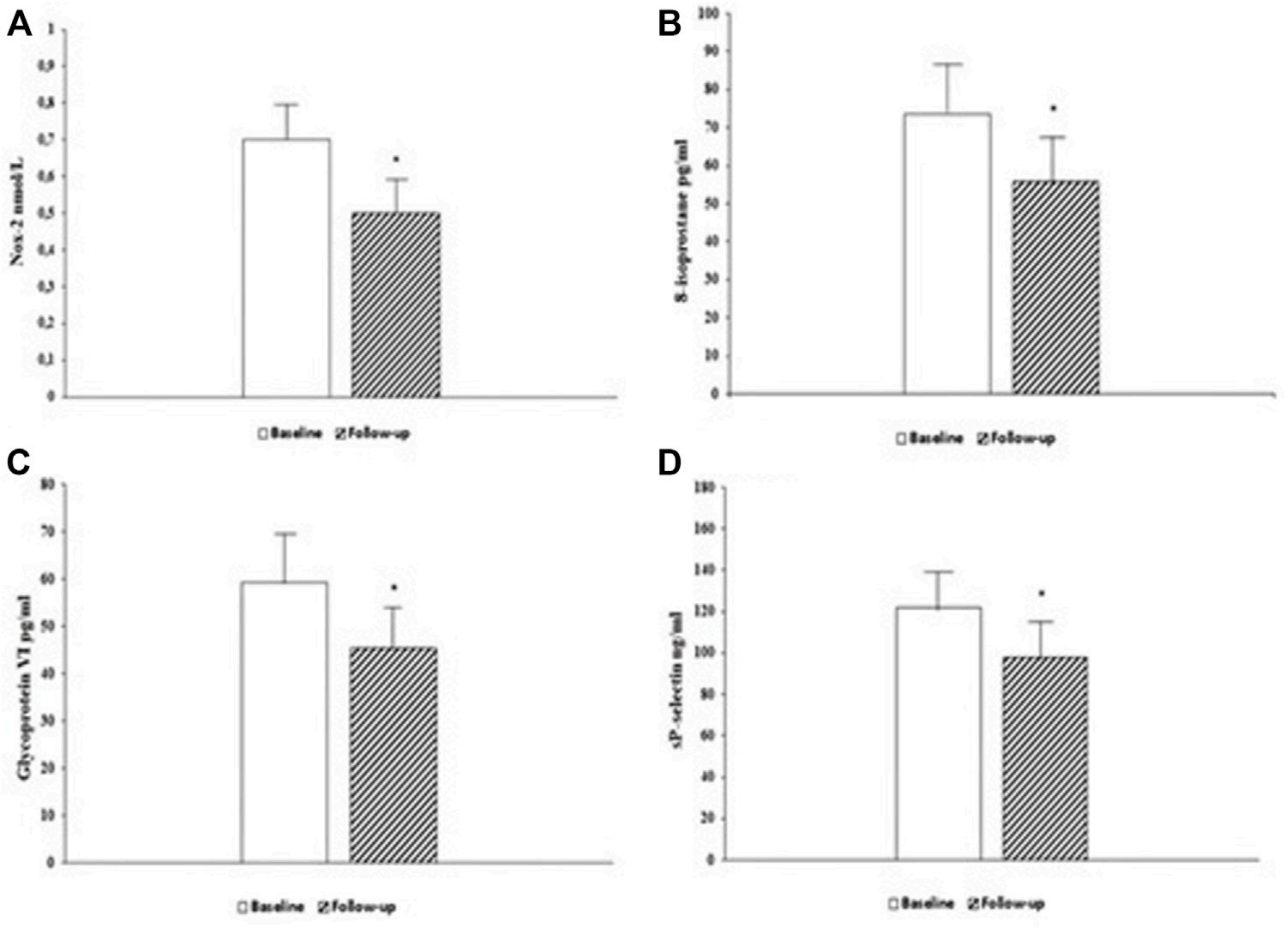
Serum levels of oxidative stress biomarker Nox-2, at baseline and 6-month follow-up **(A)**. Serum levels of oxidative stress biomarker 8-isoprostane, at baseline and 6-month follow-up **(B)**. Serum levels of platelets activity biomarker Glycoprotein VI (GPVI), at baseline and 6-month follow-up **(C)**. Serum levels of platelets activity biomarker sP-selectin, at baseline and 6-month follow-up **(D)**. Data are mean ± SD. *p < 0.0001 vs. baseline.

Likewise, oxidative stress indices such as Nox-2 (0.7 ± 0.1 vs. 0.5 ± 0.1 nmol/L, *p* < 0.0001) (Figure 1A) and 8-isoprostane (73.5 ± 10.8 vs. 55.7 ± 10.0 pg/ml, *p* < 0.0001) (Figure 1B) and platelets activation biomarkers such as GPVI (59.2 ± 12.0 vs. 45.3 ± 11.0 pg/ml, *p* < 0.0001) (Figure 1C) and sP-selectin (122.3 ± 19.6 vs. 98.1 ± 15.7 ng/ml, *p* < 0.0001) (Figure 1D) significantly improved after 6 months of treatment with Sacubitril/Valsartan.

### 3.3 Arterial stiffness and endothelial function evaluation

**FIGURE 2:**
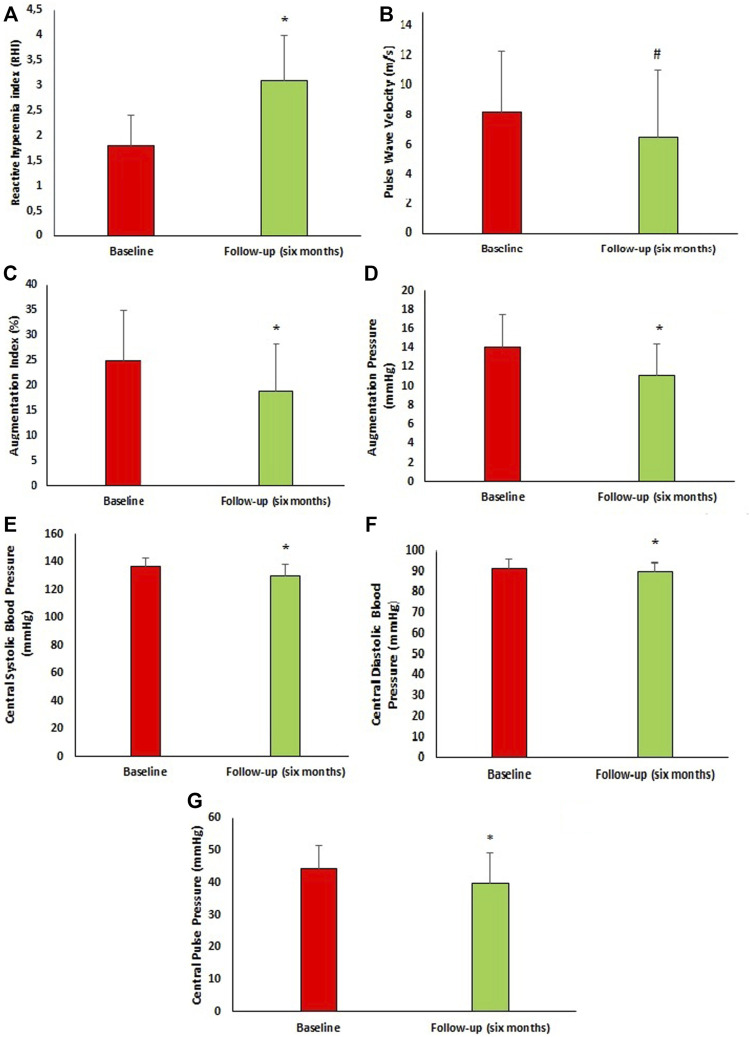
Vascular characteristics of the study population at baseline and after 6 months follow-up. **(A)** Reactive hyperemia index at baseline and after 6 months follow-up. **(B)** Pulse Wave velocity at baseline and after 6 months follow-up. **(C)** Augmentation index at baseline and after 6 months follow-up. **(D)** Augmentation pressure at baseline and after 6 months follow-up. **(E)** Central systolic blood pressure at baseline and after 6 months follow-up. **(F)** Central diastolic blood pressure at baseline and after 6 months follow-up. **(G)** Central Pulse Pressure at baseline and after 6 months follow-up. Data are mean ± SD. *p < 0.0001 vs. baseline, #*p* = 0.002 vs. baseline. (Student t-test).

**FIGURE 3:**
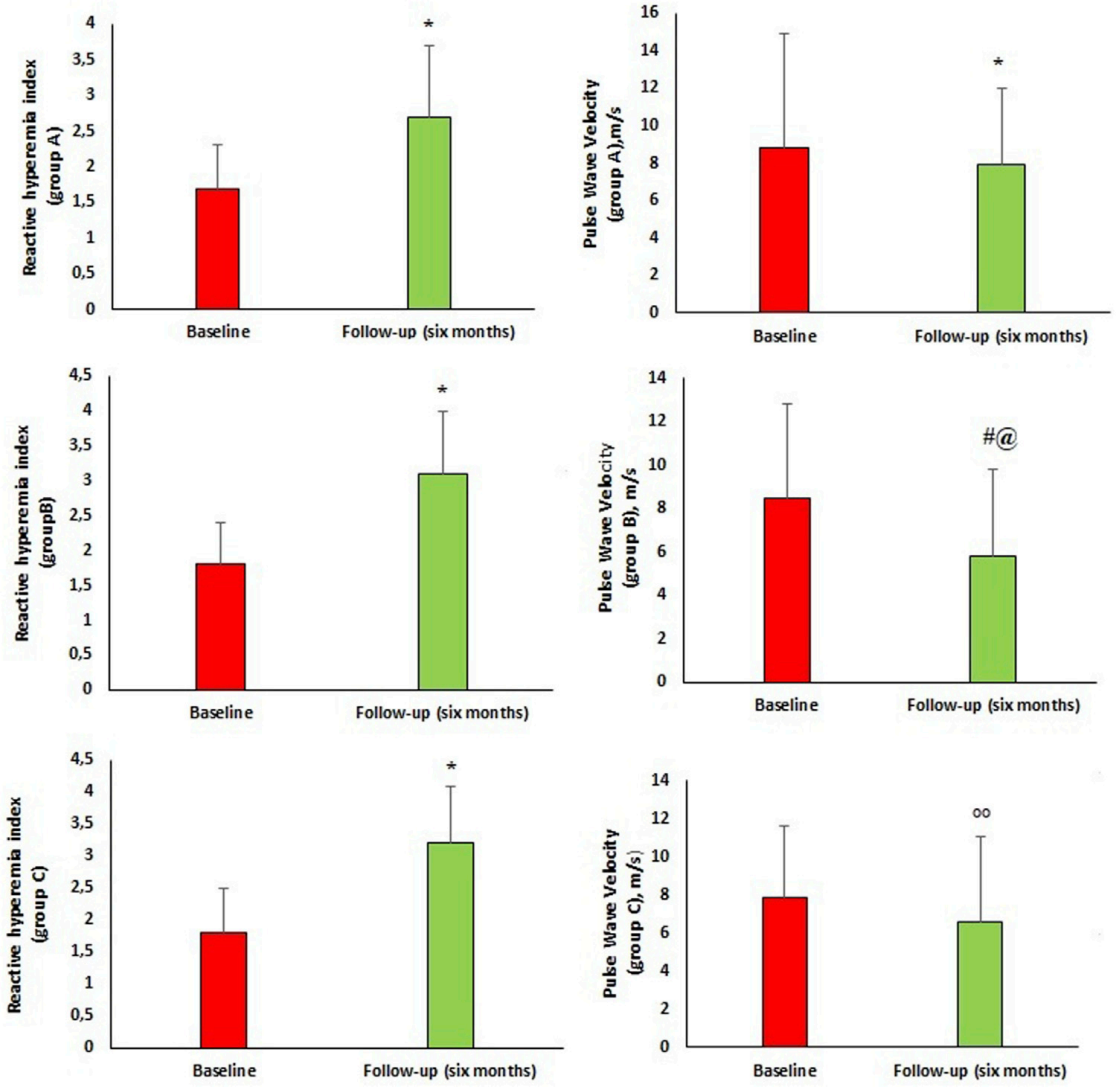
Group A, B and C are referred to the doses of sacubitril/valsartan. Group **(A)** 16 patients taking Sacubitril/Valsartan 24/26 mg bid, Group **(B)** 61 patients taking Sacubitril/Valsartan 49/51 mg bid, Group **(C)** 23 patients taking Sacubitril/Valsartan 97/103 mg bid.

Peripheral and central hemodynamic parameters at baseline and 6 months follow-up are reported in Figure 2. After 6 months of follow-up, there was significant improvement in central hemodynamic parameters, in particular there was a reduction in central SBP (c-SBP) (136.6 ± 5.9 vs. 129.9 ± 8.2 mmHg, *p* < 0.0001) and central DBP (c-DBP) (91.8 ± 4.1 vs. 89.7 ± 4.6 mmHg, *p* < 0.0001). Similarly, we observed a significant reduction in arterial stiff-ness parameters such as PWV (8.2 ± 4.1 vs. 6.5 ± 4.6 m/s, *p* = 0.002), AI (%) (24.9 ± 10.0 vs. 18.9 ± 9.4, *p* < 0.0001), AP (14.1 ± 3.4 vs. 11.1 ± 3.3 mmHg, *p* < 0.0001) and a significant improvement in endothelial function as demonstrated by RHI increase (1.8 ± 0.6 vs. 3.1 ± 0.9, *p* < 0.0001). In addition, we performed a sub-analysis in the three groups of patients taking the different dosages of Sacubitril/Valsartan to assess changes in PWV and RHI (Figure 3). In particular, at follow-up, in the 16 patients taking the lowest dosage of Sacubitril/Valsartan (24/26, mg bid) we observed changes in PWV (*p* < 0.0001) as shown in Figure 3. In the group (61 pts) taking the intermediate dosage (49/51 mg bid), PWV varied from 8.5 ± 4.3 to 5.8 ± 4.0 m/s, *p* = 0.001 and RHI from 1.8 ± 0.6 to 3.1 ± 0.9, *p* < 0.0001. Finally, in the 23 patients taking the highest dosage (97/103 mg bid), PVW decreased from 7.9 ± 3.7 to 6.6 ± 4.5 m/s, *p* = 0.024; while RHI increased from 1.8 ± 0.7 to 3.2 ± 0.9, *p* < 0.0001. Finally, we performed a sub-analysis according to the different comorbidities to assess changes in PWV and RHI. In particular, in patients with T2DM (66 pts) we observed changes in PWV from 8.1 ± 3.9 to 6.5 ± 4.5 m/s, *p* = 0.018 and in RHI from 1.8 ± 0.6 to 3.1 ± 0.9, *p* < 0.0001; in patients without T2DM (34 pts): PWV from 8.7 ± 4.6 to 6.6 ± 4.8 m/s, *p* = 0.005 and of the RHI from 1.7 ± 0.7 to 3.1 ± 0.9, *p* < 0.0001; in AF pa-tients (32 pts) we observed changes in PWV from 7.6 ± 3.4 to 6.3 ± 4.7 m/s, *p* = 0.030; and of the RHI from 1.8 ± 0.6 to 3.0 ± 0.8, *p* < 0.0001; in patients without AF (68 pts): PWV from 8.8 ± 4.5 to 6.7 ± 4.6 m/s, *p* = 0.007; and of RHI from 1.8 ± 0.6 to 3.1 ± 1.0, *p* < 0.0001. In dyslipidaemic patients (92 pts), PWV varied from 8.4 ± 4.1 to 6.3 ± 4.2 m/s, *p* < 0.0001; and RHI from 1.8 ± 0.6 to 3.1 ± 0.9, *p* < 0.0001; in non-dyslipidaemic (8 pts) PWV from 9.5 ± 7.7 to 6.7 ± 4.2 m/s, *p* = 0.02; and RHI from 1.6 ± 0.7 to 2.6 ± 1.0, *p* < 0.033. In patients with CKD (27 pts), PWV varied from 8.9 ± 5.1 to 7.6 ± 5.5 m/s, *p* < 0.0001; and RHI from 1.6 ± 0.6 to 3.0 ± 1.0, *p* < 0.0001; in non-CKD patients (73 pts) PWV from 8.0 ± 3.7 to 6.2 ± 4.2 m/s, *p* < 0.0001; and RHI from 1.8 ± 0.6 to 3.1 ± 0.9, *p* < 0.0001.

### 3.4 Correlation analysis

A linear correlation analysis was performed to test the correlation between Δ values of oxidative stress markers (Δ8-isoprostane, ΔNox-2) and different covariates express as Δ variation between baseline and follow-up. Δ8-isoprostane was directly correlated with ΔHOMA (r = 0.249, *p* = 0.013), ΔlnIL-6 (r = 0.390, *p* < 0.0001), and Δhs-CRP (r = 0.209, *p* = 0.037); furthermore ΔNox-2 resulted directly correlated with ΔHOMA (r = 0.261, *p* = 0.009) and Δhs-CRP (r = 0.254, *p* = 0.011) (Figure 1D). Moreover a linear correlation analysis was performed to assess the association between vascular parameters (ΔPWV and ΔRHI), as dependent variables and different covariates expressed as Δ variation (Table 4). ΔPWV was directly correlated with Δ8-isoprostane (r = 0.305, *p* = 0.002), ΔNox-2 (r = 0.262, *p* = 0.009), ΔSp-selectin (r = 0.292, *p* = 0.003), ΔGPVI (r = 0.319, *p* = 0.001), Δhs-CRP (r = 0.341, *p* = 0.001), ΔLogIL-6 (r = 0.381, *p* < 0.0001), ΔLogTNF-α (r = 0.231, *p* = 0.001), ΔHOMA (r = 0.371, *p* < 0.0001). ΔRHI was inversely correlated with Δ8-isoprostane (r = −0.460, *p* < 0.0001), ΔNox-2 (r = −0.329, *p* = 0.001), ΔSp-selectin (r = −0.358, *p* < 0.0001), ΔGP6 (r = −0.401, *p* < 0.0001), Δhs-CRP (r = −0.391, *p* < 0.0001), ΔLogTNF-α (r = −0.213, *p* = 0.033), ΔLogIL-6 (r = 0.237, *p* = 0.017), ΔHOMA (r = −0.269, *p* = 0.007).

**TABLE 4:** Correlation analysis between RHI and PWV, expressed as Δ variation between baseline and 6 months follow-up, and different covariates.

	ΔRHI	ΔPWV
	r/p	r/p
Δ8-isoprostane	−0.460/<0.0001	0.305/0.002
ΔNox-2	−0.329/0.001	0.262/0.009
ΔsP-selectin	−0.358/<0.0001	0.292/0.003
ΔGPVI	−0.401/<0.0001	0.319/0.001
Δhs-CRP	−0.391/<0.0001	0.341/0.001
ΔLogTNF-α	−0.213/0.033	0.231/0.001
ΔLogIL-6	−0.237/0.017	0.381/<0.0001
ΔHOMA	−0.269/0.007	0.371/<0.0001
Δe-GFR	0.242/0.015	−0.237/0.018

Abbreviations: GPVI, Glycoprotein VI; hs-CRP, highly sensitive c-reactive protein; interleukin-6; TNF-α, Tumour necrosis factor α; HOMA, homeostasis model assessment.

Variables reaching statistical significance correlation in linear regression analysis were inserted in a stepwise multivariate linear regression model to identify independent predictors of Δ PWV and Δ RHI (expressed as Δ variation). ΔLogIl-6 was the stronger predictor of ΔPWV, justifying 13.6% (*p* < 0.0001), Δhs-CRP and ΔHOMA added respectively another 6.9% (*p* < 0.0001) and 5.6% (*p* < 0.0001) of its variation. Furthermore, Δ8-isoprostane was the major predictor of ΔRHI explaining 20.4% of its variation, Δhs-CRP added another 8.4% (*p* < 0.0001) (Table 5).

**TABLE 5:** Stepwise multiple regression analysis on RHI and PWV, expressed as Δ variation between baseline and 6 months follow-up.

ΔRHI			
	Partial *R* ^2^	Total *R* ^2^	P
Δ8-isoprostane	20.4%	20.4%	<0.0001
Δhs-CRP	8.4%	28.8%	<0.0001
ΔPWV			
	Partial R^2^	Total R^2^	P
ΔLogIL-6	13.6%	13.6%	<0.0001
Δhs-CRP	5.6%	19.2%	<0.0001
ΔHOMA	6.9%	26.1%	<0.0001

Abbreviations: hs-CRP, highly sensitive c-reactive protein; interleukin-6; HOMA, homeostasis model assessment.

## 4 Discussion

To the best of our knowledge, this is the first study to demonstrate the possible effect of Sacubitril/Valsartan, a combination of Ang II receptor blocker and neprilysin inhibitor, on endothelial function and arterial stiffness in HF patients.

The endothelium is a monolayer of cells covering the inner surface of blood vessels and acts as structural and functional barrier between vessels wall and blood. It is known that the endothelium is involved in the control of thrombosis and thrombolysis, leukocytes adhesion and platelets aggregation, and the regulation of vascular tone by balancing vasoactive substances production (Marti et al., 2012) (El-Battrawy et al., 2016a) (El-Battrawy et al., 2019b).

In particular, endothelium-derived NO is released in response to physical stimuli, hormones, and platelet-derived substances and it induces vascular relaxation and platelet function inhibition.

Endothelial dysfunction, characterized by a shift in the properties of the endothelium toward impaired vasodilation promoting a pro-inflammatory and pro-thrombic state, is associated with reduced NO bioavailability (Elkayam et al., 2002). It has a crucial role in the pathogenesis of CVD and it is correlated to HF predicting major clinical events in this setting of patients (Zuchi et al., 2020) (El-Battrawy and Akin, 2022).

Notably, endothelium-dependent vasodilation is impaired in patients with both reduced and preserved HF (Kubo et al., 1991; Ramsey et al., 1995; Morgan et al., 2004; Katz et al., 2005). In particular, the dysfunction of vascular endothelium can be demonstrated in several vascular beds. According with this, several studies high-lighted a significant reduction in endothelial-dependent vasodilation in peripheral arteries in patients with chronic HF (Kubo et al., 1991; Ramsey et al., 1995; Morgan et al., 2004; Katz et al., 2005).

Of interest, endothelial dysfunction can be found not only in patients with ischemic but also non-ischemic HF, affecting non-coronary vascular beds (Husmann et al., 2015; Cassano et al., 2020). Moreover, a study conducted by Maio et al. (2021), demonstrated that impaired endothelium-dependent vasodilatation is able to predict development of HF in hypertensive patients, allowing to hypothesize its role in the CV continuum, from hypertension to clinical events.

The endothelial dysfunction observed in HF patients is amply justified by increased formation of superoxide radicals and other oxidant substances in the vascular beds. The increase in oxidative stress promotes a direct inactivation of NO thus favouring a reduced endothelium dependent vasodilation (Paolocci et al., 2001; Zuchi et al., 2010; Breitenstein et al., 2017). Furthermore, previous studies demonstrated that endothelial function and NO bioavailability can affect arterial stiffness, an independent predictor value for CVD (Westergren et al., 2016). Multiple mechanisms potentially underlie in-creased arterial stiffness including both functional and structural vascular wall changes so as collagen deposition, smooth muscle hypertrophy and changes in endothelial per-meability. Neurohormonal activation, in particular RAAS, that characterizes HF patients has been linked to endothelial dysfunction and increased arterial stiffness, by diminishing vascular elasticity, by modulating extracellular matrix growth and by inducing smooth muscle cells proliferation (Incalza et al., 2018).

Present data obtained from this longitudinal, one-center study, demonstrated that six months treatment with Sacubitril/Valsartan had positive effects on the improvement of endothelial dysfunction and arterial stiffness in a consistent cohort HFrEF symptomatic patients, as well as a significant improvement of clinical, biochemical and haemodynamic parameters, confirming previous evidence (Armentaro et al., 2021; Pelaia et al., 2022). In accordance with a study conducted by Abumayyaleh et al. (2021), conducted in patients HFrEF and stratified by age, our enrolled population is defined older (>65 years old) (mean age 69.7 ± 7.7), moreover, a further stratification of the population according to gender (82 male and 18 female) showed that there was no difference in age (male mean age 69.6 ± 7.7 and female mean age 70.4 ± 7.8, *p* = 0.663). Therefore, the entire population of our study can be considered older.

The novelty of our study is the significant betterment of inflammatory profile, as evidenced by the decreased values of IL-6, TNF-α, hs-CRP, and a reduction of oxidative stress and platelets activation biomarkers, after 6 months of treatment with Sacubitril/Valsartan; all these mechanisms may account for the improvement in vascular function.

In details, data obtained from our study showed a significant decrease in biomarkers of oxidative stress at follow-up, and a significant correlation with vascular function parameter (ΔRHI and ΔPWV). The decrease in oxidative stress levels could be due to the ability of Sacubitril/Valsartan to block angiotensin II receptor, as well as the reduction of inflammatory indices. In fact, it is known that, in patients suffering from HF, Angiotensin II, pro-inflammatory cytokines and other pathological stimuli such as hypercholesterolemia and hyperglycaemia, significantly stimulate the expression and activity of NAD(P)H oxidase (Incalza et al., 2018). In particular, Nox-2 isoform of NAD(P)H contributes to reduced bioavailability of NO and plays a crucial role in vascular disease, promoting inflammation, endothelial dysfunction and consequently arterial stiffness, *via* excessive production of ROS (Drummond et al., 2011; Ismaeel et al., 2018).

Furthermore, previous studies demonstrated that oxidative stress might lead to platelets hyperactivity; in fact, ROS over production is relevant for increase in important markers of platelets activation including sP-selectin, glycoprotein VI (GPVI) and, thromboxane B2 (TXB2) (Fuentes et al., 2019). Platelets activation has a crucial role for CVD development; in fact, platelets constitute atherothrombotic plaques, whose rupture is capable of triggering arterial thrombosis and infarction. The key role that platelets activity has in thrombotic events and in the onset and progression of atherosclerosis, might at least in partly justify the association between arterial stiffness and CVD. Our results demonstrated a significant reduction in sP-selectin and GPVI levels, after six months of Sacubitril/Valsartan treatment, indicating a reduction in platelets activity; the correlation analysis between ΔRHI, ΔPWV and platelets activity biomarkers may also explain the improvement in endothelial function and arterial stiffness observed in our enrolled patients. Reduction in platelet activation in our cohort of patients is also related to improvement in inflammatory profile; an *in vitro* study conducted by De Biase et al. (2003) proved that TNF-α may promote platelets oxidative stress, by activating NADPH enzyme oxidase. Furthermore, Pignatelli et al. (2005) demonstrated that, in HF patients, circulating levels of TNF-α may account for platelet hyperfunction because TNF-α is able to activate platelets through stimulation of the arachidonic acid pathway.

In addition, our results demonstrated a significant improvement in metabolically in-dices such as FPG, FPI, HbA1c and HOMA, and these data are in agreement with our previous evidence (Armentaro et al., 2022). Many studies demonstrated that hyperglycemia triggers vascular damage by creating imbalance between NO availability and ROS accumulation (Kaur et al., 2018; Cassano et al., 2022). Moreover, chronic hyperglycaemia injures vascular wall by many cellular mechanisms, including an increased production of intracellular advanced glycation end products (AGEs), raised expression of AGE receptors (RAGE) and ligands, activation of protein kinase C (PKC), augmented polyol and hexosamine flux and over activation of the hexosamine pathway. In our study, a linear correlation analysis showed a significant and inversely correlation between ΔRHI, ΔPWV, and ΔHOMA, indicating that with the reduction of the HOMA index there is an improvement in the endothelial function parameter and in arterial stiffness. This is clinically relevant because HOMA index is more strongly associated with CVD than glucose or insulin concentration, in non-diabetic patients (Gast et al., 2012). Furthermore, a study conducted by Westergren et al. demonstrated that HOMA index was associated with endothelial dysfunction in non-diabetic patients with myocardial ischemia and that high values of HOMA index correlated with higher IL-6 levels (Westergren et al., 2016).

**FIGURE 4:**
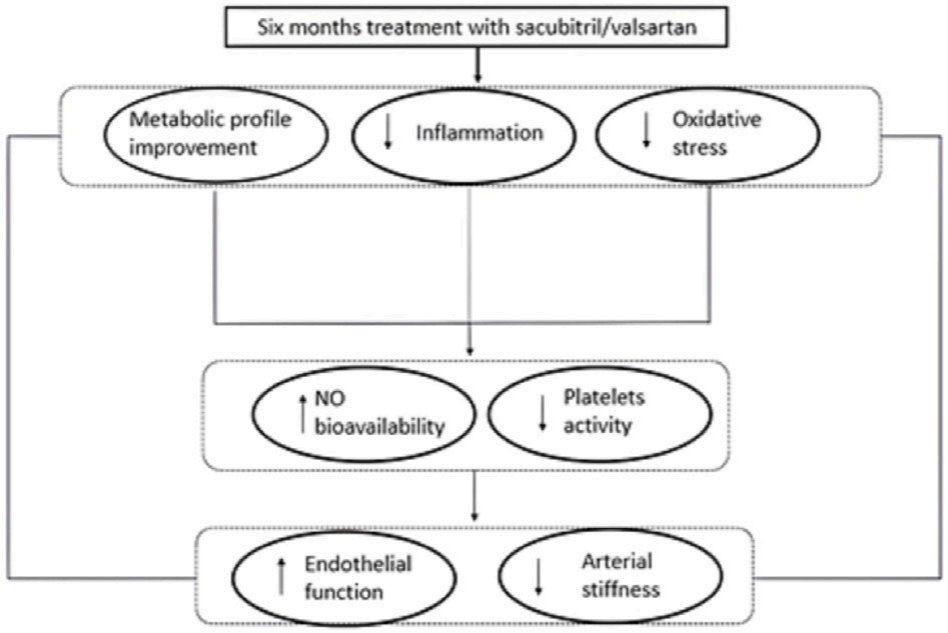
Graphical illustration of plausible pathophysiological mechanisms underlying the positive effect of the treatment with Sacubitril/Valsartan on endothelial function and arterial stiffness in HFrEF patients. NO = nitric oxide.

The increased levels of IGF-1 observed in our cohort of patients, at six months follow-up, confirm the amelioration of metabolic profile; according with this lower IGF-1 levels have previously been shown to correlate with endothelial dysfunction (Sesti et al., 2014), so the increased levels of IGF-1 observed in our study may also explain the improvement of vascular function. In addition, also the reduction of uric acid levels observed in our enrolled patients, may contribute to improve endothelial dysfunction and arterial stiffness. In fact, it is known that hyperuricemia has negative effects on CVD, by reducing NO bioavailability and promoting oxidative stress and vascular inflammation, thus favouring endothelial dysfunction (Cassano et al., 2020). Moreover, uric acid may negatively affect insulin-sensitivity by modulating the intracellular insulin signalling pathway. All these mechanisms are responsible for subclinical organ damage, indicating that higher value of uric acid are associated with arterial stiffness (Perticone et al., 2013). Finally, it is important to remark that Sacubitril/Valsartan significantly improved vascular parameters, in particular endothelial function and arterial stiff-ness, known predictors of CV outcome. This improvement, as evidenced by the regression analysis, is linked to the improvement of oxidative stress and inflammation indices. Notably, RHI and PWV follow-up values were comparable to those of newly diagnosed hypertensive patients without diabetes or CV complications (Sciacqua et al., 2012; Cassano et al., 2021). Similarly, the indicators of inflammation, in particular hs-CRP, and of oxidative stress significantly improved with Sacubitril/Valsartan to such an extent that they were comparable to those of normotolerant hypertensive patients (Cassano et al., 2022) (Figure 4).

## 5 Conclusion

In conclusion, the present study strengthens scientific evidence supporting the use of Sacubitril/Valsartan in HFrEF patients and confirms its beneficial effects on hemodynamic, clinical, biochemical and metabolic profile. Moreover, the novelty of our study is that six months treatment with Sacubitril/Valsartan is able to improve endothelial dysfunction and arterial stiffness in HFrEF patients, likely through the reduction of oxidative stress, platelet activation and inflammation biomarkers circulating levels with the improvement of cardio-metabolic burden.

## Data Availability

The original contributions presented in the study are included in the article/Supplementary Material, further inquiries can be directed to the corresponding author.
